# Necessity of Pure Air

**Published:** 1878-05

**Authors:** 


Thus it is, that after writing, or reading, or sewing, in one position for a long time, and the whole body feels tired, we get up, stretch the body, draw a full deep breath and walk across the room a few times, there is a feeling of rest and refreshment comes over us which is most agreeable. Why? Because the full breath distends the air-cells, straightens the blood vessels, the blood passes onward, presenting itself as it passes, to the life giving influences of the air in the freshly and fully distended air vessels. What madness it is, what deliberate suicide, to repress these yearnings of our instincts for the life-giving agencies which a beneficent Providence has thrown around us with such bounteous profusion: the Pure Air of Heaven!

But how does the blood become thus impure at the right side of the heart, before it goes for renovation to the lungs? There are two sources of impurity. A barrel of the purest water will be sadly defiled, if taken to the attic, and every floor in the house is washed with it, down to the cellar. The blood starts from the lungs pure and clean, it goes through the whole frame, washing out as it goes along, all the particles of our body which have died since the last visit; for we are always dying, reader! Particles which have subserved their uses, and having answered the great end of their creation, must be swept away as the cinders from the grate or the ashes from the hearth. Thus the blood, so pure but two and a half minutes before, is now loaded with offal, and is deposited in the heart, the great Clearing House of the body. So this body of ours is swept out, is washed clean every two minutes and a half of our existence. Like a magnificent steam engine requiring the constant attendance of the engineer, who if he does his duty, is all the time cleaning and oiling, so as to keep it in perfect working order, so is our body.

Does not the reader see, then, that not only is the want of full breathing a cause of impure blood, but that if the air he breathes is not pure when first breathed, it can no more unload the blood of its impurities as perfectly as it ought to have been done, than dirty water can wash a garment clean? You, who habitually breathe an impure, that is, confined air, for all confined air is impure, are a moral suicide. Hurry then, from your bed-chamber the instant of rising; hoist the windows of your sitting apartments, fling wide open your doors, divers times daily, even in the coldest weathers, and let out the death, instead of drawing it into your own system, to fester, and corrupt and rot you.

The other great cause of blood impurity at the right side of the heart, is the following:

We eat to live. What we eat is turned into blood, the object of that blood is two-fold. First, to keep us warm. Second, to repair the wastes of the system. Washing these wastes away in the manner we have named, is a matter of secondary importance, as to the blood; it is rather an incidental work. To keep us warm and to repair, these are First Things.

The process of converting food into blood is as follows:

After entering the stomach, it is converted into a sweetish whitish fluid in about two hours, when it is gradually passed out of the stomach along the intestines down to the vent of the system, receiving as it passes out of the stomach, drop by drop, the bile from the liver. In about four hours after eating an ordinary meal the stomach is empty, and in another hour or two we begin to get hungry. Opening into the stomach and all along the intestines, there are multitudes of open-mouthed tubes whose office it is to absorb, or withdraw what is real nutriment, from the passing mass of food, its essence. Some of it is ready to be withdrawn while in the stomach, other portions only become ready at various points along the intestinal passages, some only at the end. Thus it is that some elements of food are not converted into nutriment until long after having passed out of the stomach. These diminutive tubes convey their contents towards the great central tube of the system, just as the various springs, rivulets, creeks, &c., of any of our great rivers, flow together until all are united in one magnificent stream, which itself is finally emptied into the boundless sea. The heart is the great receiving sea of the myriads of nutriment-bearing channels of the human system. This nutrimental material enters the heart at the same time that another great river pours its contents into it; that river of blood which started from the heart a very few minutes before, and having washed out the body, delivers the defiled mass into the heart again to be renovated, refined, vivified. So that at any moment, the right side of the heart is industriously receiving two different kinds of fluid, the washings out of the body, and the imperfect nutrient material for blood, just as the Mississippi and Missouri pour very different waters together at their uniting point, soon mingling, however, into one homogeneous stream. The impure blood and the nutrient material soon coalesce, commingle and enter the lungs for purification, thoroughly mixed together. There meeting with the air, the nutrient fluid is in an instant converted into pure blood, and in the same instant of time, are the washings of the system converted into blood equally pure, by having had all its impurities abstracted at a breath. Thus we see, that in reality, our food does not become living, actual blood, until it has entered the lungs and been exposed to the life-giving influences of the air therein; hence we see that if air has not its life, that is its purity, it is utterly impossible for the food we have eaten to receive that finishing stroke which makes it real, perfect blood. And if not perfect, the system is imperfectly fed, and debility and disease are inevitable.

